# High-Throughput Peptide Derivatization toward Supramolecular Diversification in Microtiter Plates

**DOI:** 10.1021/acsnano.0c05423

**Published:** 2021-02-15

**Authors:** Yiyang Lin, Matthew Penna, Christopher D. Spicer, Stuart G. Higgins, Amy Gelmi, Nayoung Kim, Shih-Ting Wang, Jonathan P. Wojciechowski, E. Thomas Pashuck, Irene Yarovsky, Molly M. Stevens

**Affiliations:** † Department of Materials, Department of Bioengineering and Institute of Biomedical Engineering, Imperial College London, Exhibition Road, London SW7 2AZ, United Kingdom; ‡ State Key Laboratory of Chemical Resource Engineering, Beijing Laboratory of Biomedical Materials, Beijing University of Chemical Technology, Beijing 100029, China; § School of Engineering, RMIT University, Melbourne, Victoria 3001, Australia; ∥ Department of Medical Biochemistry and Biophysics, Karolinska Institutet, SE-171 77 Stockholm, Sweden

**Keywords:** self-assembly, peptide, combinatorial synthesis, Suzuki−Miyaura cross coupling, high-throughput screening

## Abstract

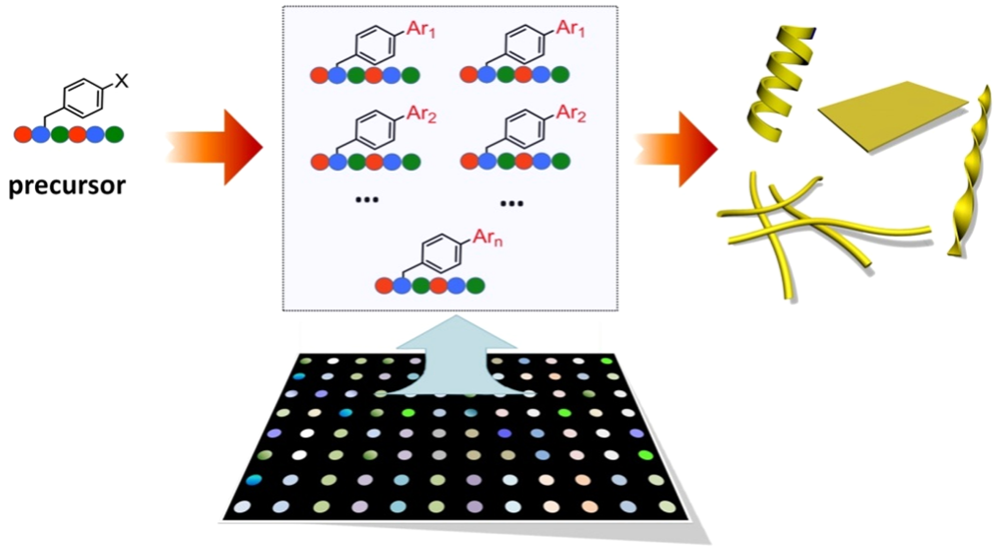

The evolution of life on earth eventually leads to the emergence of species with increased complexity and diversity. Similarly, evolutionary chemical space exploration in the laboratory is a key step to pursue the structural and functional diversity of supramolecular systems. Here, we present a powerful tool that enables rapid peptide diversification and employ it to expand the chemical space for supramolecular functions. Central to this strategy is the exploitation of palladium-catalyzed Suzuki–Miyaura cross-coupling reactions to direct combinatorial synthesis of peptide arrays in microtiter plates under an open atmosphere. Taking advantage of this *in situ* library design, our results unambiguously deliver a fertile platform for creating a set of intriguing peptide functions including green fluorescent protein-like peptide emitters with chemically encoded emission colors, hierarchical self-assembly into nano-objects, and macroscopic hydrogels. This work also offers opportunities for quickly surveying the diversified peptide arrays and thereby identifying the structural factors that modulate peptide properties.

Diversity and complexity are two main features of biological systems resulting from the evolution of life on earth over long periods of time. Pursuing the structural diversification of molecular systems in a laboratory is an important step to develop functional materials with a broad range of properties. One example is polypeptide diversification through sequence engineering with the combinations of 20 canonical amino acids, which provides a variety of structures and functions across different dimensions.^1−8^ Additional complexity and added benefits (*e*.*g*., molecular fluorescence, biological activity, site-specific modification, and strengthened aggregation propensity) can be achieved through synthetically incorporating unnatural amino acids or functional groups.^9−17^ However, such a chemical approach to obtain peptide diversification is usually associated with tedious organic synthesis. Moreover, it is acknowledged that subtle chirality/sequence/structure modifications can cause changes in the peptide conformation, association energy landscape, and binding affinity.^18−28^ In this context, rapidly screening or predicting the behavior of peptide arrays in an efficient manner is also essential for expanding peptide diversity and accelerating their applications as innovative soft materials.^29^ At the molecular level, phage display employs the genetic amplification of bacteriophage coat proteins to build libraries for identifying specific recognition sequences. Research at the supramolecular level is more challenging since a combination of molecular interactions involving multiple weak forces need to be taken into consideration. One intriguing example is the supramolecular amplification of short peptides that are capable of forming a wide range of nano-objects.^30−35^ To this end, computational tools (*e*.*g*., coarse-grained molecular dynamics) have also been developed to study the association and supramolecular behaviors of short peptide libraries.^36−38^ Experimentally, Ulijn *et al*. reported the production of a peptide library by continuous enzymatic condensation, hydrolysis, and transacylation of small dipeptide fragments, which allows the peptide sequence space to be searched for supramolecular structures.^39^ Recently, a fully automated, flow-based approach to solid-phase polypeptide synthesis was reported, which accelerated peptide design aiming for high-throughput peptide screening.^40,41^

To explore the vast possibility of peptide properties and uncover the underlying structure–function relationships, an efficient chemical tool capable of expanding peptide repertoires without time-consuming batch synthesis will be of great utility. In this work, we report a strategy to direct high-throughput peptide derivatization by utilizing a palladium-catalyzed Suzuki–Miyaura cross-coupling reaction in aqueous solution, which allows for the molecules to be synthetically accessible in one step under mild conditions without compromising on the library diversity or complexity. We show the possibility of fusing a library of commercial arylboronates to short peptide precursors, either at the N-terminus or on the peptide backbone, and thus diversifying peptide structures in a highly efficient way. Using this platform, we demonstrate the general utility to discover unnatural peptide sets and identify the chemical elements determining their behaviors such as microscopic self-assembly and macroscopic hydrogelation as well as chemically encoded fluorescent nanostructures.

## Results and Discussion

### Peptide Diversification and Supramolecular Amplification

**Figure 1:**
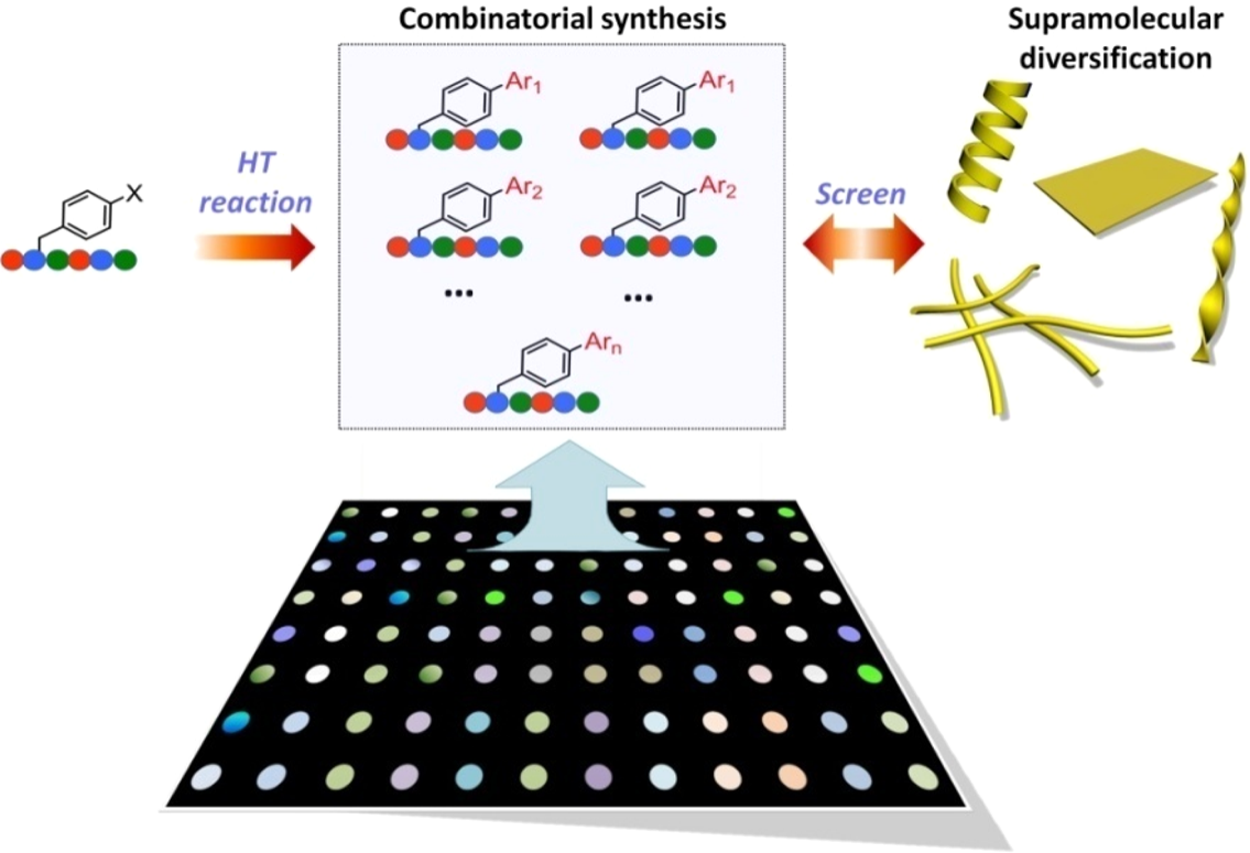
Workflow for exploration of the chemical space for peptide properties in microtiter plates. High-throughput (HT) synthesis of a peptide library was achieved by reacting an aryl halide peptide (precursor) with an array of arylboronates, allowing for the rapid screening of the structure–property relationship in supramolecular systems in low-volume microtiter plates. The multicolor pattern in plate wells represents the tunable emission colors of Suzuki peptide products as well as their self-assembled nanostructures.

Key to our high-throughput screening is the synthesis of a diversified peptide library *via* Suzuki–Miyaura cross-coupling. Previously, this palladium-catalyzed reaction has served as an efficient tool for the site-selective conjugation of proteins and the *in vitro* modification of genetically engineered cell membranes.^42,43^ Herein, we achieved combinatorial synthesis of peptide analogs by coupling a set of commercial arylboronates to aryl halide peptide precursors (Figure 1). The tolerance of the reaction to water and oxygen, the high coupling efficiency, and the versatility make the Suzuki–Miyaura coupling ideally suited for directing *in situ* peptide diversification and simultaneous library screening in a low-volume well plate.

**Figure 2:**
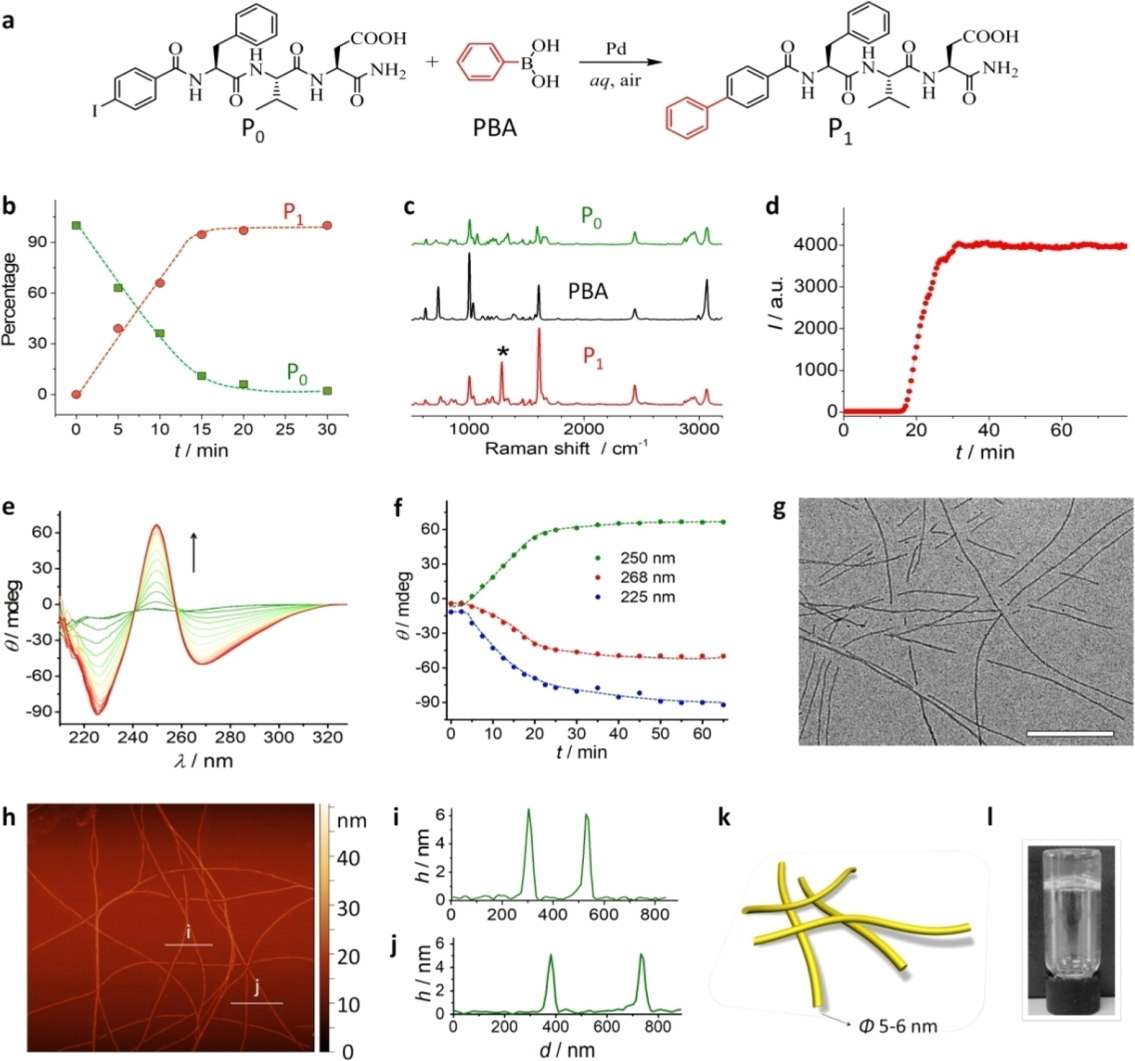
Suzuki–Miyaura cross coupling directed tripeptide synthesis and supramolecular amplification. (a) Tripeptide diversification mediated by Suzuki–Miyaura coupling reactions, where the precursor (**P**_**0**_) is converted into a biaryl product (**P**_**1**_) by reacting with PBA. (b) HPLC results showing the percentage conversion of **P**_**0**_ to **P**_**1**_. (c) Raman spectra showing the formation of biaryl product **P**_**1**_ where the peak at 1284 cm^–1^ corresponds to the inter-ring C–C bond (indicated by the asterisk). (d–f) Kinetic study of Suzuki coupling reaction-induced peptide self-assembly: (d) ThT assay; (e) CD spectra; and (f) CD intensity. (g) TEM and (h) tapping mode AFM images showing the fibril formation after the Suzuki–Miyaura reaction. (i–k) Height profiles across two fibrils in AFM image (h) suggest the thickness to be 5–6 nm. (l) Hydrogel formation after Suzuki–Miyaura reaction ([**P**_**0**_] = 5 mM). Scale bars: (g) 500 nm and (h) 1 μm.

To validate the *in situ* peptide diversification, we designed a tripeptide precursor (**P**_**0**_) containing phenylalanine, valine, aspartic acid, and *p*-iodobenzoyl group at the N-terminus (Figure 2a). In the presence of sodium palladium chloride (Na_2_PdCl_4_) as a catalyst, **P**_**0**_ efficiently reacted with a series of arylboronates (structural modulator) *via* Suzuki–Miyaura cross coupling in aqueous solution and transformed into the corresponding biaryl derivatives. Depending on the structure of the arylboronate coupling partner, the biaryl products exhibit varied supramolecular properties. We initially verified the cross-coupling reaction between **P**_**0**_ and phenylboronic acid (PBA) *via* mass spectroscopy, high-performance liquid chromatography (HPLC), and Raman spectroscopy. Mass spectroscopy confirmed the formation of biaryl product **P**_**1**_ (*m*/*z* = 557) and the disappearance of **P**_**0**_ (*m*/*z* = 607) after reaction (Figure S1). HPLC showed the reaction was complete within 20 min with >95% conversion (Figure S2 and Figure 2b). Raman spectra of the reaction product also showed the appearance of a peak at 1284 cm^–1^, corresponding to the stretching mode of inter-ring C–C bond of biphenyl motif (Figure 2c).^44^

We then studied the supramolecular self-assembly of tripeptide products from Suzuki–Miyaura cross coupling by fluorescence assays, transmission electron microscopy (TEM), atomic force microscopy (AFM), and circular dichroism (CD). The precursor **P**_**0**_ is relatively hydrophilic with a critical aggregation concentration (c*) of ∼2.0 mM (Figure S3), meaning the peptide exists in the monomeric or nonaggregated state below this concentration. Upon the addition of palladium salt and PBA, the thioflavin T (ThT) fluorescence in **P**_**0**_ solution (1.0 mM) significantly increased over time after incubating for ∼20 min and reached a plateau after ∼30 min, indicating the existence of a hydrophobic, viscous environment resulting from tripeptide aggregation that restricts ThT intramolecular rotation (Figure 2d). In a control experiment without Na_2_PdCl_4_, the ThT fluorescence remained unchanged, indicating the indispensible role of catalyst and biphenyl formation (Figure S4). We also used the Nile Red fluorescence assay to confirm that reaction-driven tripeptide self-assembly led to the formation of a hydrophobic domain (Figure S5). Further studies with CD demonstrated the amplification of supramolecular chirality upon conjugation as self-assembly was induced (Figure 2e,f). CD signals enhanced at 225, 250, and 268 nm and reached a plateau after 30 min, which suggests that tripeptide self-assembly was completed within 30 min, in agreement with the results of ThT assay (Figure 2d). Notably, CD signal amplification occurred at ∼5 min, while the fluorescence emission from ThT and Nile red began to increase at ∼18 min. This suggests **P**_**1**_ self-assembly underwent a process involving nucleation–oligomerization–fibrillation, where the CD onset corresponded to the molecular nucleation, and the fluorescence increase was indicative of tripeptide fibrillation. TEM and AFM (Figure 2g–k) showed uniform fibrils with 5–6 nm thickness (5.3 nm on average, Figure S6), which is equal to the length of two peptide molecules, indicating that the fibrils have bilayer structures. Although the precursor **P**_**0**_ shows a relatively weak propensity toward self-assembly, as can be seen from the higher critical aggregation concentration (2.0 mM), the tripeptide can form intermolecular hydrogen bonds and aromatic interactions which contribute to the stability of the β-sheets and are indispensable for driving fibrillation of Suzuki coupling products. In a control experiment where the precursor **P**_**0**_ was replaced by 4-iodophenylacetic acid or 4-iodobenzoic acid, we did not observe the phenomenon of reaction-driven fibrillation.

At higher tripeptide concentrations (>5 mM), the cross coupling could eventually lead to the formation of a self-supporting hydrogel (Figure 2l). The versatility of the Suzuki–Miyaura cross coupling is highlighted by the use of the brominated tripeptide as the precursor (Figure S7) and Pd nanocubes as the catalyst (Figure S8). Compared to palladium ions, Pd nanoparticles possess higher stability under physiological conditions and lower toxicity for biology relevant applications and, therefore, represent a promising catalytic system for future studies.^45,46^

### Molecular Dynamics Simulations

**Figure 3:**
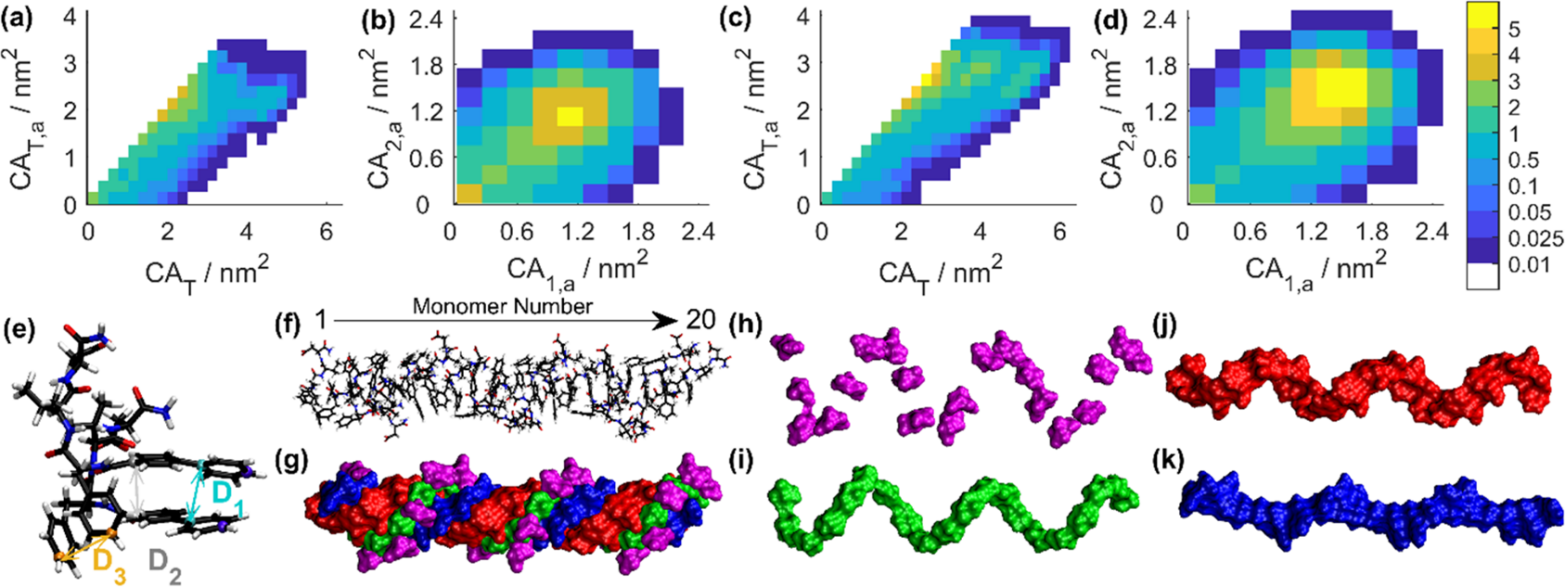
MD simulation. Population maps of CA_T,a_ against CA_T_ for (a) **P**_**0**_ and (c) **P**_**1**_ and of CA_2,a_ against CA_1,a_ for (b) **P**_**0**_ and (d) **P**_**1**_ with the color bar indicating the percentage of simulation time (note the nonlinear increments). For clarity, the 53.2% and 31.9% of simulation time for **P**_**0**_ and **P**_**1**_, respectively, where CA_T_ = 0 nm^2^ is excluded from population maps. (e) Dimer configuration identified as capable of forming a helical fiber with *D*_1_ and *D*_2_ being the distance between carbon atom pairs shown in silver and teal, respectively, in the aromatic mutation region and *D*_3_ being the distance between the phenyl ring carbon atoms shown in orange in each monomer. (f) All atom snapshot of the protofiber. (g–k) van der Waals surface of the protofiber components: (g) all; (h) Asp (magenta); (i) Val (green); (j) Phe (red); and (k) the aromatic core (blue).

We rationalize the different self-assembly behaviors of the tripeptides before and after Suzuki–Miyaura cross coupling by molecular dynamics (MD) simulations. The interaction of two tripeptide monomers in water was investigated for both **P**_**0**_ and **P**_**1**_ to determine: the degree of association, the regions of the tripeptides responsible for association, and whether a dimer of **P**_**0**_ or **P**_**1**_ could be identified which might act as a nucleation seed for a higher order structure. The amount of the solvent accessible surface area (SASA) of a monomer in direct contact with the other monomer (identified as “covered”) was calculated (CA_*i*_, where *i* = 1 or 2) and used to assess the degree of association between the tripeptide monomers. The total covered area, defined as CA_T_ = CA_1_ + CA_2_, was found to be 0 nm^2^ for 53.2% and 31.9% of the simulation time for **P**_**0**_ and **P**_**1**_, respectively, indicating a stronger association between **P**_**1**_ monomers. This is in agreement with the lower c* of **P**_**1**_ (∼0.3 mM) relative to that of **P**_**0**_ (∼2.0 mM, Figure S3). CA_*i*_ was separated into two regions: (1) the “aromatic region” including the side chain of phenylalanine along with the appended region, CA_*i*,a_, and (2) the “peptide region” which included the amide backbone of phenylalanine along with the valine and aspartic acid residues, CA_*i*,p_ (Figures S9 and S10). Two-dimensional (2D) population maps of the covered SASA for various regions of the tripeptides were generated to determine which regions of the monomers are influential in driving association (Figure 3a–d, Figures S9 and S10). For both the **P**_**0**_ and **P**_**1**_ tripeptides, these maps show that CA_T_ is correlated with CA_T,a_ (Figure 3a,c). The population maps of CA_2,a_ against CA_1,a_ exhibit a single high-density region centered above (1 nm^2^, 1 nm^2^), suggesting a strong association between the aromatic regions of the monomers (Figure 3b,d). Conversely, 2D population maps show that the highest density of CA_T_ exists when CA_T,p_ = 0 (Figures S9a and S10a). Furthermore, the population maps of CA_2,p_ against CA_1,p_ show that the peptide region is not likely to interact with the peptide region of the other monomer. The most populous regions of these maps align with either axis, indicating an absence of cooperation between the peptide regions of the monomers with regards to association (Figures S9b and S10b). The limited coverage of the peptide region without aromatic association again supported the experimental conclusion that it is the aromatic component which drives the self-assembly process.

To investigate whether the dimers formed in the **P**_**1**_ system had the potential to act as nucleation seeds for a higher order structure, the configurations of peptide dimers were studied (Figure 3e, Figure S11). Given the importance of the aromatic region for tripeptide association, the relationship between the distance (*D*_1,2,3_) between three different carbon atom pairs and CA_T,a_ was examined (Figure 3e and Figure S11). *D*_1_ and *D*_2_ are distances between carbon atoms in the aromatic region of **P_1_**, as indicated in Figure 3e. When *D*_1_ and *D*_2_ are small, there is, not unsurprisingly, a high degree of association between the aromatic regions of the tripeptide molecules (Figure S11b,c). For 27.7% of simulation time, both *D*_1_ and *D*_2_ are <0.6 nm, while CA_T,a_ is >2.5 nm^2^, that is, dimers fall within the regions defined by the dashed white lines in Figure S11a,b, indicating that the dimers have the mutated aromatic region of the two **P**_**1**_ monomers aligned with one another. *D*_3_, the distance between the phenylalanine carbon in each tripeptide as indicated in Figure 3e, is associated with high CA_T,a_ (Figure S11c). When *D*_3_ is <8 Å (a more relaxed (*i*.*e*., distant) contact criterion), dimers are observed to fall within the regions defined the dashed white lines in Figure S11a–c for 12.6% of the simulation time, indicating that dimers with all three aromatic rings of the monomers in close proximity and high contact are frequently observed, making them highly likely to be the nucleation seeds for a higher order structure.

To distinguish between various dimer configurations that fell within the distance criteria discussed above, the angle between the rings of the phenylalanine and the aromatic mutation of **P**_**1**_ were calculated (as indicated in Figure S11e). 2D population maps of these angles show four distinct regions (Figure S11d). Examination of the dimer configurations within each of these four regions and within the cutoff criteria for *D*_1_, *D*_2_, and *D*_3_ revealed that the dimer formed when both angles were centered around 120° yielded a seed configuration with potential fibrillation capacity. This dimer, identified within the dashed lines in Figure S10a–d, was observed for 5.4% of total simulation time. The feature of this dimer configuration is that both tripeptide molecules have the same conformation and the association of a third tripeptide in the same conformation either above or below (see Figure 3e for reference) preserves that site for the association of another tripeptide, thus enabling this tripeptide dimer to potentially act as a nucleation seed for fibrillation.

To test the stability of such a fiber, a 20 monomer protofiber was constructed computationally from this dimer seed (Figure 3f–h, S12). The fiber was stable for the duration of the simulation and exhibited a measurable helical pitch and periodicity of ∼2.2 nm and 6 monomers per turn (Figure S12). The stable helical fiber has an aromatic core with only 27% of the aromatic mutated region exposed to the solvent in the formed fiber relative to the tripeptide monomer (Table S1). The chiral arrangement of the aromatic groups is supported by experimentally observed CD signals (Figure 2e). Similarly, both phenylalanine and valine, two hydrophobic amino acids, have only 42.2% and 33.5% solvent exposure in the fiber compared to the monomer in solution (Table S1). In contrast, the hydrophilic aspartic acid residue has 77% of the available SASA exposed to water in the fiber compared to the monomer (Table S1). In addition to the hydrophobic association of the fiber core, it was observed that the protofiber was stabilized by hydrogen bonding between the nitrogen of the valine backbone and the terminal oxygen of phenylalanine of the adjacent tripeptide monomer in the fiber (Figure S16). Based on a geometric definition of hydrogen bonds with a distance cutoff of 0.35 nm and an angle cutoff of 25°, there are, on average, 13.3 ± 2.0 hydrogen bonds between these two atoms along the 20 monomer protofiber. For comparison, based on the same definition, there are on average 5.5 ± 2.0 hydrogen bonds within the fiber other than those between these two atoms. Thus, the stable fiber structure formed by the identified tripeptide seed minimizes the unfavorable interaction of hydrophobic regions with water and is stabilized with a robust hydrogen-bond pattern along the spiral, while not sacrificing any of the favorable interactions aspartic acid has with water.

### Chemical Space Exploration for Tripeptide Self-Assembly

**Figure 4:**
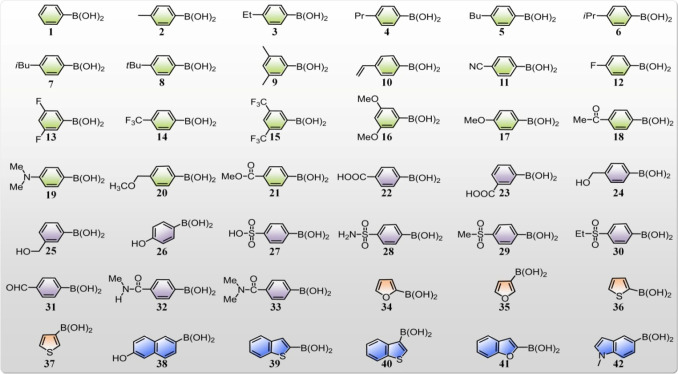
A library of aryl boronates with varied hydrophobicity, polarity, charge groups, and aromaticity were used to couple to the tripeptide precursor (**P**_**0**_) *via* Suzuki–Miyaura cross-coupling reaction. The aromatic groups are labeled with different colors: hydrophobic phenyl groups (green), phenyl groups with polar substitution (violet), five-member rings (furyl or thienyl group, orange), and large π-conjugated groups (blue). Tripeptide products derived from **22**–**35** cannot aggregate into ordered nanostructures.

The use of Suzuki–Miyaura cross coupling allows us to facilely develop a peptide library in which their properties could be explored independently. Here, supramolecular self-assembly of tripeptide products was directly verified in each case by ThT and TEM. In particular, we selected 42 commercially available arylboronates with varied hydrophobicity/hydrophilicity, aromaticity, charge states, and polarity and coupled them with the iodinated tripeptide precursor **P**_**0**_. Generally, the fusion of a hydrophobic aryl group to **P**_**0**_ promoted tripeptide fibrillation (Figure 4, Figure S13). This effect could be enhanced by either adding methylene (−CH_2_−) or methyl (−CH_3_) groups (*e*.*g*., **2**–**10**), a cyano group (**11**), or substituting aromatic hydrogen with fluorine (*e*.*g*., **12**–**15**). In contrast, the incorporation of anionic groups (−COOH and −SO_3_H, **22**, **23**, and **27**) suppresses extensive self-assembly by introducing electrostatic repulsion. This effect could be reversed by shielding of the carboxylate by esterification (−COOCH_3_, **21**) to remove the negative charge at physiological pH, while this phenomenon was not observed by amidation of the carboxylate (**32** and **33**). This difference is likely due to the higher hydrophobicity of esters than the corresponding amides, as determined from the logarithm of the 1-octanol–water partition coefficient, log*P*.^47^ Similarly, the deactivating effect of the sulfonate group (−SO_3_H, **27**) could not be compensated by simply removing negative charge (**28**–**30**), due to the high polarity of the sulfonyl group (−SO_2_−) when compared to the corresponding carbonyl group (C=O). This can be rationalized by comparing the relative polarity of dimethyl sulfoxide (*E*_*T*_^*N*^ = 0.444) to acetone (*E*_*T*_^*N*^ = 0.355).^48^

When comparing aldehyde (−CHO) and ketone (−COCH_3_) functionalized tripeptides, both of which bear a polar carbonyl group (C=O), the biaryl derivative with ketone **18** aggregated into nanofibrils, while the aldehyde **31** did not. This indicates that the addition of a methyl substituent sufficiently alters tripeptide self-assembly, highlighting the role of subtle molecular changes in influencing supramolecular properties. Polar groups like hydroxyl (−OH, **26**) and hydroxylmethyl (−CH_2_OH, **24** and **25**) were found to inhibit tripeptide association, while the methoxy group substitution (**16**, **17**, and **20**) restores the propensity for tripeptide fibrillation. Notably, even those tripeptides containing the same atomic composition but different architectures display significant differences in self-assembly. For instance, arylboronates with hydroxylmethyl (−CH_2_OH) substituents (**24** and **25**) act as structural deactivators, while the methoxy group substitution (−OCH_3_, **17**) is a fibrillation promoter. This difference is reasonable considering the fact that alcohol groups are known to be more polar than the corresponding ether.

The conjugation of heteroaromatic furans (**34** and **35**) to **P**_**0**_ did not induce tripeptide aggregation; however, the analogous sulfur containing thiophenes (**36** and **37**) were found to trigger the fibrillation. This is reasonably ascribed to the higher aromaticity of thiophene over furan,^49^ which is believed to contribute to enhanced π–π stacking that promotes tripeptide association. To stress the contribution of molecular aromaticity, we compared two aryl groups of naphthol (**38**) and phenol (**26**) and observed that the tripeptide fused with the naphthol group had a much stronger propensity to undergo fibrillation than the latter. Further experiments with aryl boronates containing large aromatic groups including benzo[*b*]thiophene-2-boronic acid (**39**), benzo[*b*]thiophene-3-boronic acid (**40**), benzo[*b*]furan-2-boronic acid (**41**), and 1-methyl-5-indolylboronic acid (**42**) again highlight the central role of aromaticity (Figure S12).

**Figure 5:**
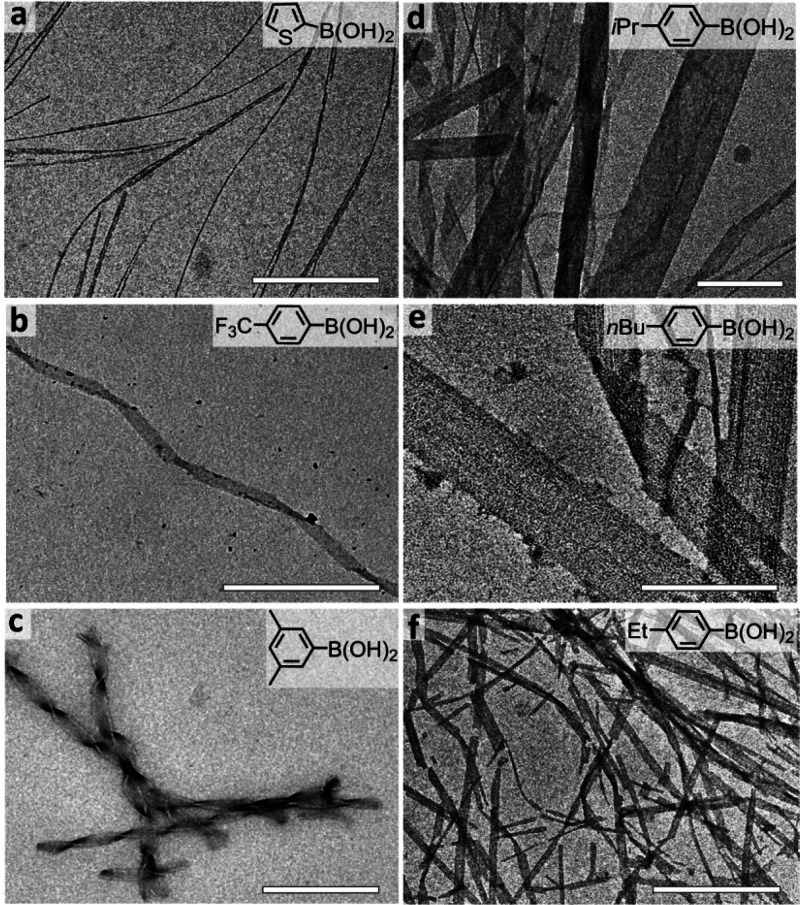
Representative TEM images showing the diversified self-assembly nanostructures from Suzuki–Miyaura cross coupling between the precursor **P**_**0**_ and arylboronates: (a) thin fibrils, (b) helical ribbons, (c) twisted ribbons, (d) wide planar ribbons, (e) laminated ribbons, and (f) thin ribbons. More examples are presented in Figure S13. Scale bars: (a, b, d, f) 500 nm and (c, e) 250 nm.

Next, we investigated whether varying the arylboronate coupling partner could modulate the self-assembly morphology. Indeed, supramolecular structures including nanofibrils (Figure 5a), helical (Figure 5b), and twisted (Figure 5c) ribbons, rigid planar ribbons (Figure 5d), and laminated (Figure 5e) and thin ribbons (Figure 5f) were all observed when the precursor was conjugated with varied arylboronates. More examples can be seen from TEM images in Figure S13. The tendency to form helical or twisted nanostructures in several of the products may be due to the known ability of aromatic groups, including the adjacent Phe residue, to induce such structures.^50,51^ It is noticed that as the organoboronate increases in steric bulk, the width of the resultant peptide nanofibrils increases and 2D nanostructures become energetically favored, as highlighted by the planar ribbons resulting from the coupling of arylboronates **4**–**8** (Figure 5 and Figure S13). We speculate that hydrocarbon tails (*e*.*g*., propyl and butyl groups) can contribute to strong interpeptide aggregation in the direction perpendicular to hydrogen bonds and, therefore, promote tripeptide association in these two directions.

In summary, peptide modification *via* Suzuki–Miyaura cross coupling strongly influenced tripeptide assembly. We analyzed a variety of structural factors (*e*.*g*., aromaticity, polarity, hydrophobicity, and charge effects) that govern peptide self-assembly and demonstrated that subtle changes in the appended aryl group can significantly affect the free energy associated with the self-assembly process. The arylboronates can be classified into four groups based on their structural features and performances. Arylboronates with polar groups (*e*.*g*., −OH, −SO_2_NH_2_, −SO_2_CH_3_, and −CH_2_OH) are deactivators that suppress tripeptide aggregation by tuning the balance of hydrophobicity and hydrophilicity. Charged arylboronates with −COOH and −SO_3_H are even stronger deactivators due to the incorporation of additional charge repulsion that curtails tripeptide association. In contrast, the addition of hydrophobic or aromatic substitutions (*e*.*g*., phenyl, thiophenyl, naphthenyl groups, or fluorine substitution) provides additional short-peptide interactions. We calculated the partition coefficient between octanol and water (reported as log*P*) of the aryl groups in the modified peptides (Table S2) and correlated this parameter with tripeptide fibrillation. There is a trend for the attachment of aryl groups with a high log*P* (>1.5) to **P**_**0**_ to promote tripeptide self-assembly.

### Diversification of Peptide Emitters

**Figure 6:**
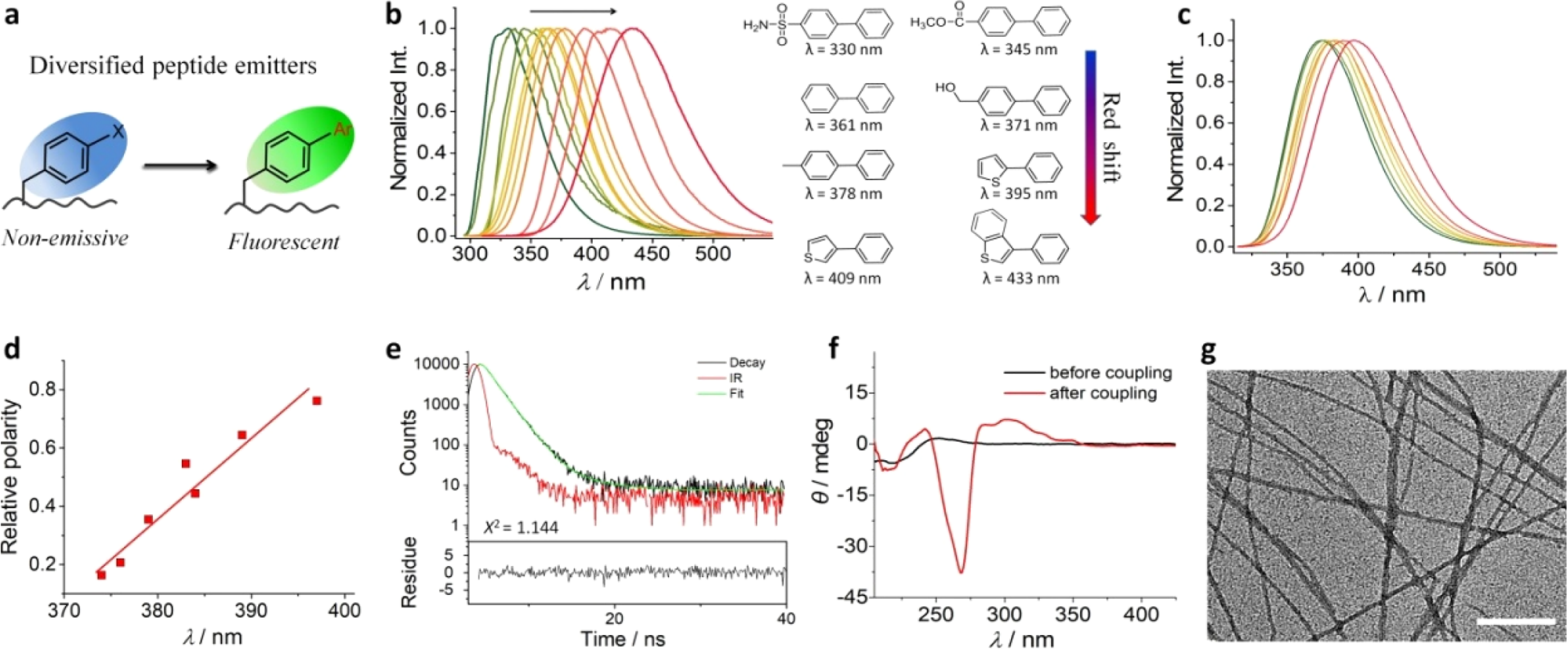
(a) Schematic illustration of combinatorial synthesis of tripeptide emitters from a nonemissive peptide precursor and a variety of arylboronates. (b) Fluorescence spectra of tripeptide products derived from arylboronates (from left to right: **28**, **14**, **21**, **22**, **1**, **12**, **24**, **2**, **36**, **37**, and **40**). The corresponding biaryl products as well as their maximum wavelengths are also listed. (c) Fluorescence spectra of tripeptide product **P**_**39**_ in different organic solvents (left to right: 1,4-dioxane, THF, NMP, dimethyl sulfoxide, isopropanol, ethanol, and methanol). (d) Correlation of solvent relative polarity^48^ with the maximum emission wavelength of the tripeptide products. (e) Time-dependent fluorescence decay of **P**_**39**_ indicating the fluorescence lifetime of 1.39 ns. (f) CD spectra showing the chirality amplification after cross coupling with the arylboronate **39**. (g) A representative TEM image showing the formation of nanofibrils from tripeptide **P**_**39**_. Scale bar: 200 nm.

We went on to demonstrate that the combinatorial synthesis of the tripeptide library enabled the discovery of color-tunable peptide emitters. In biology, green fluorescent protein (GFP) is widely exploited as a fluorescent probe.^52^ GFP is made up of 238 amino acid residues in a single polypeptide chain, and its fluorescence originates from a post-translational modification of the tripeptide -Ser^65^-Tyr^66^-Gly^67^ to generate an imidazole-based chromophore. Previously, the aggregation of dipeptides into highly ordered structures has been shown to induce quantum effects and thereby intrinsic fluorescence.^53^ Here, we show that high-throughput synthesis of emissive tripeptides can be achieved through the Suzuki–Miyaura cross coupling of nonfluorescent boronates and precursor **P**_**0**_ (Figure 6a). The generation of structurally diverse biphenyls extends π conjugation and therefore enhances molecular fluorescence. Depending on the electron-withdrawing/-donating groups appended to the arylboronate, the maximum fluorescence emission peak could be tuned between 330 and 433 nm (Figure 6b). For example, the tripeptide conjugated with a phenyl group (**P**_**1**_) has an emission peak at 361 nm, while peptides appended with aryl motifs bearing electron-withdrawing sulfonyl (−SO_2_NH_2_, **28**) or carbonyl (−COOCH_3_, **21**) groups exhibit emission peaks at ∼330 and 345 nm, respectively. Tripeptides appended with electron-donating hydroxymethylphenyl (−PhCH_2_OH, **24**) or methylphenyl (−PhCH_3_, **2**) groups show emission maxima at 371 and 378 nm, respectively. Increasing the aromaticity by using large aryl rings such as benzo[*b*]thiophene-3-boronic acid (**40**) contributes to extension of π conjugation, such that the emission maximum further shifts to 433 nm. Importantly, this tunability can be delivered despite the use of a common phenyl iodide peptide precursor. With further diversification of the coupling partner, even greater ranges of fluorescence emission will be accessible.

The fluorescence emission of tripeptide products was found to be sensitive to changes in the local environment due to the rotation of biaryl groups along the newly formed C–C bonds (Figure 6c,d), similar to the twisted intramolecular charge-transfer effect where the solvent polarity or viscosity affects the optical property of molecular rotors.^54^ As an example, we studied the fluorescence of the tripeptide **P**_**0**_ appended with **39**. In organic solvents, the emission peak of **P**_**39**_ shifts from 374 to 397 nm (Figure 6d) as the solvent increases in polarity from 1,4-dioxane (*E*_*T*_^*N*^ = 0.164), tetrahydrofuran (*E*_*T*_^*N*^ = 0.207), *N*-methyl-2-pyrrolidone (*E*_*T*_^*N*^ = 0.355), dimethyl sulfoxide (*E*_*T*_^*N*^ = 0.444), isopropanol (*E*_*T*_^*N*^ = 0.546), ethanol (*E*_*T*_^*N*^ = 0.654), to methanol (*E*_*T*_^*N*^ = 0.762).^48^ A good correlation between the hypsochromic shift of the fluorescence spectrum and the relative polarity of the organic solvent was observed. In aqueous solution, the time-resolved fluorescence decay of **P**_**39**_ was dominated by a fast decay mode (1.39 ns, 98.31%, Figure 6e), suggesting the majority of **P**_**39**_ molecules remained nonaggregated. By using quinine sulfate (in 0.1 M H_2_SO_4_) as a standard, the quantum yield of **P**_**39**_ was measured to be 6.2%. At supramolecular level, the conjugation of **39** to **P**_**0**_ leads to chirality amplification and fibril formation through strong interpeptide stacking (Figure 6f,g).

Furthermore, the local environment-sensitive fluorescence emission (*i*.*e*., spectral shift) of Suzuki products allows us to distinguish their monomeric and aggregated states. For example, the fluorescence emission peak of the coupling product **P**_**37**_ was observed to red-shift from 370 to 382 nm as the concentration increased from 0.1 mM to 2.0 mM (Figure S14). This is because tripeptide self-assembly above CAC significantly changed its environmental properties (*e*.*g*., micropolarity and microviscosity). As such, this Suzuki product can serve as a self-reporting fluorescent probe to monitor its aggregation status.

## Conclusions

We report a high-throughput method to achieve supramolecular structures by directing *in situ* peptide diversification *via* Suzuki–Miyaura cross coupling in aqueous solution. At the molecular level, we show the combinatorial synthesis of a peptide library and the design of intrinsically fluorescent peptides with chemically encoded emission colors. At the supramolecular level, the molecular diversification provides access to the discovery of supramolecular structures (*e*.*g*., nanofibrils, flat ribbons, and twisted and helical nanoribbons) as well as the identification of structural factors (*e*.*g*., aromaticity, polarity, hydrophobicity, and charge effects) that governs tripeptide self-assembly. In addition, the *in situ* combinatorial synthesis allows for the rapid screening of structure–function relationships and supramolecular properties such as emission and conductivity (Figure S15). Taken together, this work develops a chemical discovery tool toward adaptive peptide arrays and dynamic supramolecular nanosystems that holds great promise for potential applications to complement with the computer-aided peptide designs and machine learning strategies. The kinetically controlled palladium-catalyzed reactions are expected to offer extra control over supramolecular order.

## Methods

### Reagents and Materials

Rink amide resin, 9-fluorenylmethoxycarbonyl (Fmoc)-protected amino acids, 2-(1H-benzotriazol-1-yl)-1,1,3,3-tetramethyluronium hexafluorophosphate (HBTU), and Kaiser test kit were purchased from Anaspec. Dimethylformamide (DMF), diisopropylethylamine (DIEA), and dichloromethane (DCM) were purchased from AGTC Bioproducts. All the other reagents were used as received.

### Solid-Phase Peptide Synthesis

Tripeptide precursors were synthesized using standard solid-phase peptide synthesis using Rink amide resin and Fmoc-protected amino acids. To a peptide synthesis vessle, 0.25 mmol of resin was loaded and swelled in DCM for at least 30 min. The Fmoc group was removed in 20 mL of piperidine/DMF (20:80, v/v). After that, protected amino acids were coupled to the resin by using a mixture of amino acid (1 mmol), HBTU (0.98 mmol), and DIEA (1.5 mmol) in DMF. The N-terminal protected Fmoc group was then removed using 20 v/v% piperidine in DMF solution for at least 10 min and repeated twice. 4-Iodobenzoic acid and 4-bromobenzoic acid (four times excess) were coupled to the N-terminus of tripeptide using HBTU/DIEA (3.95:6 relative to the resin). The tripeptide product was cleaved from the resin using a cleavage solution containing trifluoroacetic acid/triisopropylsilane/water (95:2.5:2.5, v/v/v). After removing excess TFA in vacuum, the crude tripeptide was precipitated by cold diethyl ether and purified on reverse phase high-performance liquid chromatography (HPLC, Shimadzu) using Phenomenex C18 Gemini NX column (150 × 21.2 mm, 5 μm particle size and 110 Å pore size). The purity of tripeptide product was confirmed by matrix-assisted laser desorption spectroscopy (Waters).

### Suzuki–Miyaura Cross Coupling Reaction

In an open air environment, 800 μL of tripeptide precursor **P_0_** (1.25 mM in 10 mM phosphate buffer, pH 7.5) was mixed with 5 μL of Na_2_PdCl_4_ (10 mM in phosphate buffer, pH 7.5). To this mixture, 195 μL of aryl boronates (7.7 mM in 10 mM phosphate buffer, pH 7.5) was added, and the solution was incubated at room temperature for the stated amount of time before characterization. The final concentrations of precursor **P_0_**, Na_2_PdCl_4_, and boronate were 1.0 mM, 0.05 mM and 1.5 mM, respectively.

### Transmission Electron Microscopy

TEM characterization was performed on a JEM 2100F (200 kV), and the images were recorded with an Orius camera. Suzuki–Miyaura cross-coupling reaction was performed as described above, and the reaction proceeded for 12 h before TEM characterization. To prepare the TEM samples, 10 μL of the tripeptide product solution was placed onto the carbon-coated copper grid (200 mesh) for 5 min. Excess solution was removed with a filter paper, and the grid was then stained with 10 μL of uranyl acetate solution (1.0 wt %). After removing the excess staining agent, the TEM grid was dried in air.

### Atomic force microscopy

AFM measurements were performed in tapping mode on an AFM 5500 microscope (Keysight technologies) in ambient atmosphere. An HQ:NSC15/Al BS tip (μmasch) was used in this work (tip radius <8 nm, force constant of 40 N/m, resonance frequency of 325 kHz).

### Raman Spectroscopy Analysis

The synthesized powders were placed on a cleaned calcium fluoride Raman substrate (Crystran Ltd., UK). Raman spectroscopy was performed on a confocal Raman microspectroscopy system (alpha 300 R+, WITec GmbH, Ulm, Germany) equipped with a piezoelectric stage (UHTS 300, WITec GmbH, Ulm, Germany). A green laser (λ_ex_ = 532 nm, WITec GmbH, Ulm, Germany) with a power of ∼35.7 mW was employed with the application of a 20×/0.4 NA microscope objective lens (EC Epiplan, Zeiss, Oberkochen, Germany). The backscattered Raman signals were collected by the spectrometer (UHTS 300, WITec GmbH, Ulm, Germany) equipped with a thermoelectrically cooled (−60 °C), charge-coupled device camera (Newton, Andor Technology Ltd., UK, Belfast), using a 100 μm low-OH silica fiber as a waveguide. The spectra were obtained in a spectral range from 0 to 3600 cm^–1^ with an integration time of ∼10.0–30.0 s. The spectra were calibrated using the standard silicon peak (∼520 cm^–1^) and normalized to the area under the curve, which removed any instrument effects and reduced the signal intensity variability between the samples. All of the data postprocessing was performed using MATLAB software (MathWorks, Inc., Natick, MA, USA).

### Thioflavin T Assay

To prepare the thioflavin T (ThT) samples, 200 μL of peptide solutions was incubated with 2 μL of ThT (1 mM in water) in a 96-well plate. The fluorescence intensity of ThT was measured on a SpectraMax M5 plate reader (λ_ex_ = 440 nm, λ_em_ = 485 nm).

### Nile Red Assay

Nile Red fluorescence assay was used to detect the formation of hydrophobic domains in peptide nanostructures. In detail, peptide solutions with a total volume of 80 μL were transferred to a 384-well plate. To each well, 2.5 μM of Nile Red stock solution in ethanol was added. Fluorescence intensity at 630 nm was measured on a SpectraMax M5 plate reader with the excitation wavelength of 575 nm.

### Circular Dichroism

CD spectra of peptide solutions were recorded on a CD spectrophotometer (Jasco-715) at room temperature. A rectangular quartz cell with the light path length of 1 mm was used, and the final spectrum was averaged from three scans taken at the range of 200–350 nm.

### Model Construction

The initial models of the **P**_**0**_ and **P**_**1**_ molecules were constructed with all amino acid residues in the β-sheet arrangement and the additional termination added using the molefacture plugin in VMD.^55^ Two peptide molecules were placed 1.5 nm apart and solvated in a 4.8 × 4.8 × 4.8 nm^3^ water box (approximately 3500 water molecules) with counterions to neutralize the system. The **P**_**1**_**(20)** fiber was built by selecting a dimer-conjugate from the association simulations which met the distance cutoff criteria to have potential for fiber nucleation, and this dimer was replicated and aligned such that distance cutoff criteria for each sequential pair of **P**_**1**_ molecules was satisfied. The structure was then solvated in a 12.8 × 12.8 × 12.8 nm^3^ water box (approximately 67,000 water molecules) with counterions to neutralize the system and then equilibrated for 40 ns in the NPT (constant number of particles, pressure, and temperature) ensemble. An all-atom representation of the **P**_**0**_ and **P**_**1**_ molecules was used with the intra- and intermolecular interactions being modeled by a combination of the CHARMM36^56,57^ and CGenFF36^58^ force fields. Water molecules were treated explicitly using the TIP3P water model.^59^

### Simulation Setup

MD simulation as implemented in NAMD (version 2.12)^60^ was used for all the work reported here. In all simulations, a cutoff distance of 12 Å was applied for nonbonded interactions with switching applied between 10 and 12 Å. Long-range electrostatic interactions were treated using the particle mesh Ewald method.^61^ A time step size of 2 fs was similarly used in all simulations, with O–H bond lengths constrained using the SHAKE algorithm.^62^ NPT simulations were undertaken using a Langevin thermostat^63^ and Langevin piston Nose–Hoover method^64,65^ to control the temperature and pressure at 298 K and 1 atm, respectively. Five runs for each of the **P**_**0**_ and **P**_**1**_ dimer association simulations were run for 300 ns each with an output frequency of 10 ps. A single 40 ns simulation was performed for the **P**_**1**_**(20)** fiber with an output frequency of 10 ps. Analysis was performed on the final 250 and 20 ns for the dimer and fiber systems, respectively, using VMD.^55^

## Supporting Information Available

Mass spectra, HPLC, CD spectra, ThT and Nile Red assay, concentration-dependent fluorescence emission, log*P* of aryl groups, TEM images, MD simulation, and conductivity measurements (PDF)

## Notes

Experimental data is available at DOI: 10.5281/zenodo.4506962. Molecular dynamics simulation data is available upon request from irene.yarovsky@rmit.edu.au.
