# Supplementary material for: High-Throughput Peptide Derivatization toward Supramolecular Diversification in Microtiter Plates
Source: ACS Nano. 2021 Feb 15;15(3):4034–44. doi: 10.1021/acsnano.0c05423 (PMC7992134; doi:10.1021/acsnano.0c05423)
Supplement: Supplementary file 1 — nn0c05423_si_001.pdf [file nn0c05423_si_001.pdf]

Supplementary Information

High-Throughput Peptide Derivatization toward Supramolecular Diversification in Microtiter Plates

Yiyang Lin,[1,2] Matthew Penna,[3] Christopher D. Spicer,[4] Stuart G. Higgins,[1] Amy Gelmi,[1] Nayoung Kim,[1] Shih-Ting Wang,[1] Jonathan P. Wojciechowski,[1] E. Thomas Pashuck,[1] Irene Yarovsky,[3] Molly M. Stevens[1,4]

[1] Department of Materials, Department of Bioengineering and Institute of Biomedical Engineering, Imperial College London, Exhibition Road, London SW7 2AZ, United Kingdom

[2] State Key Laboratory of Chemical Resource Engineering, Beijing Laboratory of Biomedical Materials, Beijing University of Chemical Technology, Beijing 100029, China

[3] School of Engineering, RMIT University, Melbourne, Victoria 3001, Australia

[4] Department of Medical Biochemistry and Biophysics, Karolinska Institutet, SE-171 77 Stockholm, Sweden

Correspondence and requests for materials should be addressed to I. Y. (irene.yarovsky@rmit.edu.au) and M. M. S. (m.stevens@imperial.ac.uk)

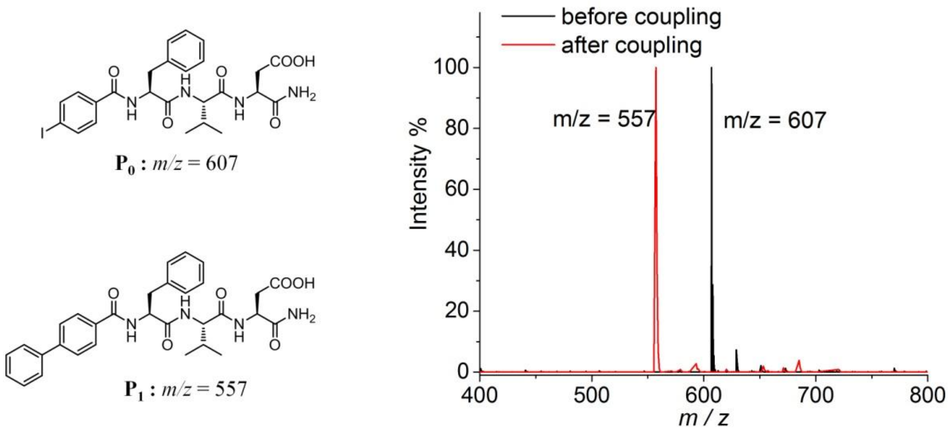

Figure S1. Mass spectra showing the tripeptide evolution from $\mathbf{P_0}$ (black, *m/z* = 607) to $\mathbf{P_1}$ (red, *m/z* = 557) *via* Suzuki-Miyaura cross coupling reaction.

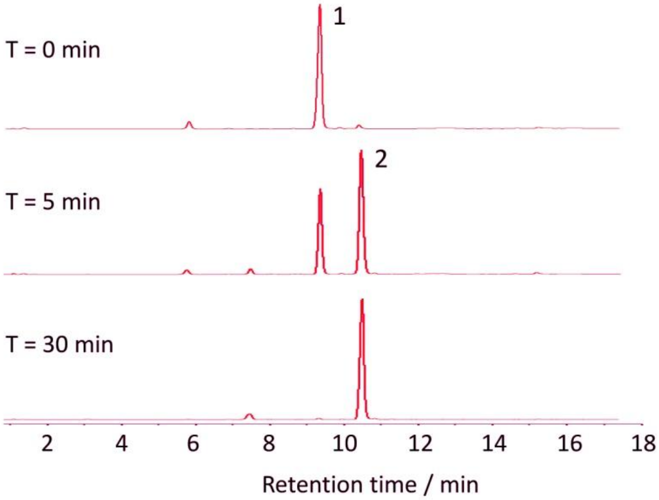

Figure S2. High performance liquid chromatography (HPLC) trace showing the tripeptide evolution *via* Suzuki-Miyaura cross coupling reaction. The solution contains 1.0 mM of $\mathbf{P_0}$, 1.5 mM of PBA, and 0.025 mM Na_2PdCl_4. Peak 1 represents $\mathbf{P_0}$ and peak 2 represents $\mathbf{P_1}$.

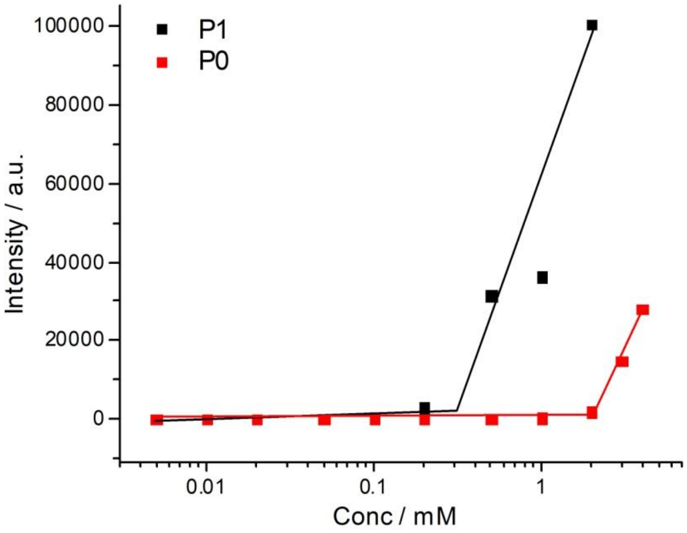

Figure S3. Fluorescence intensity of tripeptide solutions containing Thioflavin T (10 μM) was plotted against tripeptide concentrations. The critical aggregation concentration was determined to be 0.3 mM and 2.0 mM for $\mathbf{P_1}$ and $\mathbf{P_0}$, respectively. Thioflavin T is a cationic probe that increases in fluorescence upon binding to aggregated nanofibrils. The excitation wavelength was 440 nm and the emission wavelength of 485 nm.

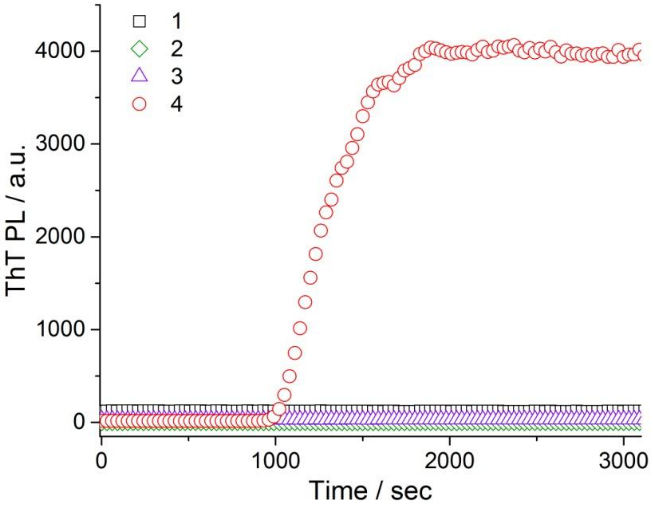

Figure S4. Fluorescence intensity of ThT in solution containing a mixture of $\mathbf{P_0}$/PBA/Na_2PdCl_4 with the concentration of: (1) 1.0 mM : 1.5 mM : 0 mM, (2) 0 mM : 1.5 mM : 0.05 mM; (3) 1.0 mM : 0 mM : 0.05 mM; (4) 1.0 mM : 1.5 mM : 0.05 mM. The concentration of ThT is 5 μM. The increase of ThT fluorescence suggests a rigid environment created by peptide aggregation (λ_{ex} = 440 nm, λ_{em} = 485 nm).

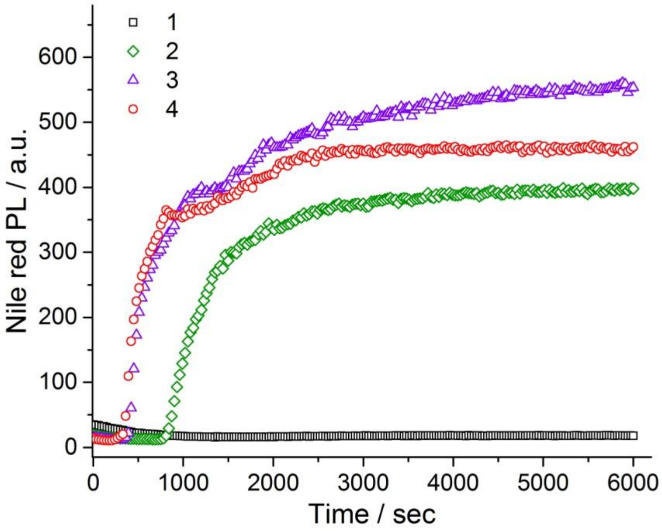

Figure S5. Fluorescence intensity of Nile Red in the solution of $\mathbf{P_0}$/PBA/Na_2PdCl_4. (1) 1.0 mM : 1.5 mM : 0 mM; (2) 1.0 mM : 1.5 mM : 0.05 mM; (3) 1.0 mM : 2.0 mM : 0.05 mM; (4) 1.0 mM : 2.5 mM : 0.05 mM. The concentration of Nile Red is 2.5 μM. The increase of Nile Red fluorescence implies the formation of hydrophobic domain of peptide nanostructures (λ_{ex} = 575 nm and λ_{em} = 630 nm). The increase in PBA concentration accelerated the fibrillation process of tripeptide product in aqueous solution. However, high concentration (i.e., 2.5 mM, Curve 4) of PBA might interfere the tripeptide self-assembly and lower the saturated fluorescence of Nile Red.

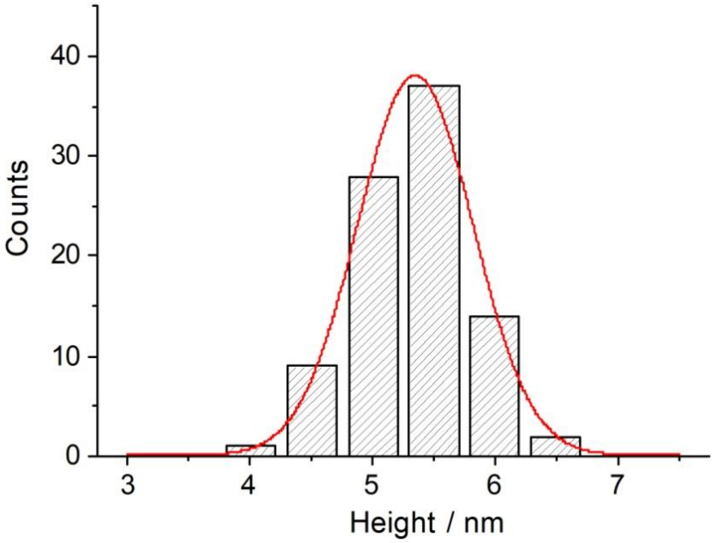

Figure S6. Atomic force microscopy height profiles of nanofibrils formed in the mixture of $\mathbf{P_0}$/PBA/Na_2PdCl_4 (1.0 mM : 1.5 mM : 0.05 mM). AFM imaging was conducted after Suzuki-Miyaura cross coupling reaction for 12 h.

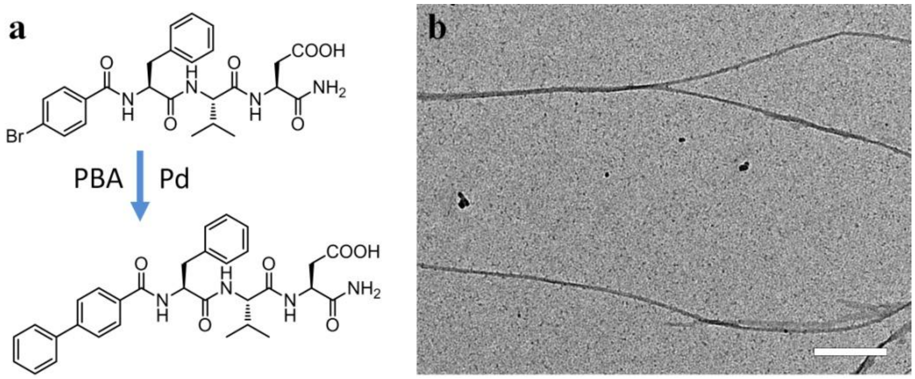

Figure S7. (a) Suzuki-Miyaura cross coupling reaction converts a brominated peptide (precursor) into its biaryl product. (b) A negative-stained TEM image showing the formation nanofibrils from the peptide system containing 1.0 mM of tripeptide precursor, 1.5 mM of phenylboronic acid (PBA), and 0.05 mM of Na_2PdCl_2. TEM imaging was conducted after Suzuki-Miyaura cross coupling reaction for 12 h. Scale bar: 250 nm.

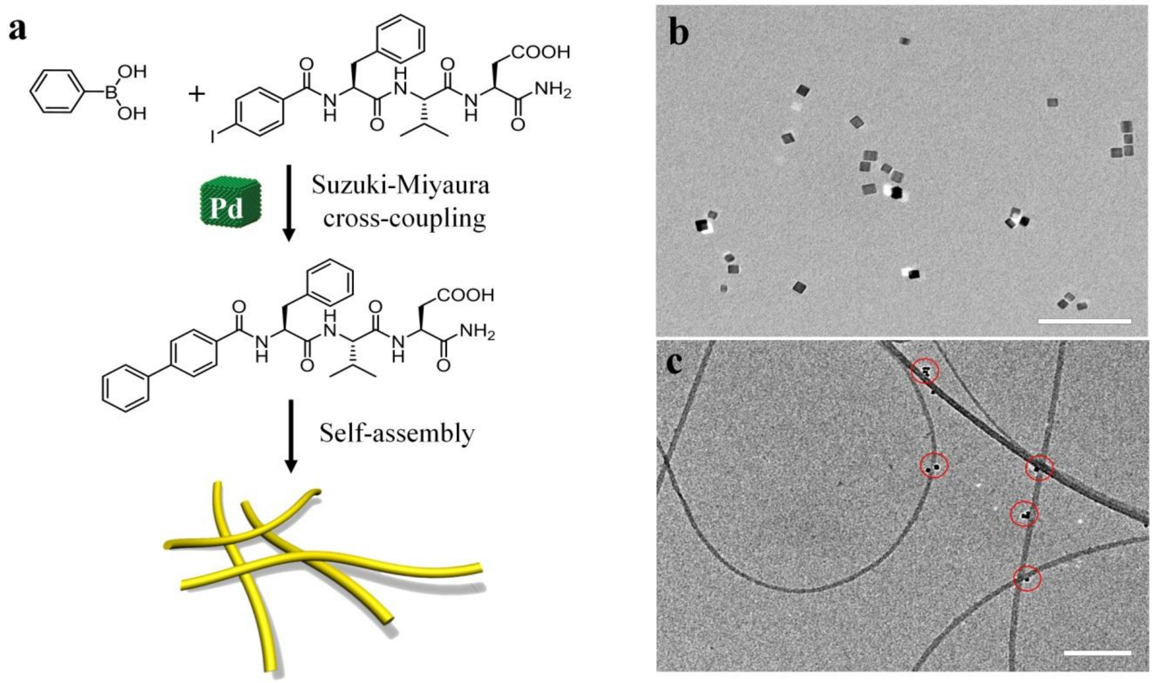

Figure S8. (a) Palladium nanocube-triggered Suzuki coupling between $\mathbf{P_0}$ and phenyl boronic acid in aqueous solution. TEM images showing (b) the cubic morphology of polyvinylpyrrolidone (PVP)-coated palladium nanoparticles and (c) well-defined self-assembled peptide fibrils after Suzuki coupling. Interestingly, the Pd nanocubes remain non-aggregated after reaction and PdNPs are observed to align on the surface of peptide fibrils, suggesting the important role of PdNPs in catalyzing cross-coupling reaction. TEM imaging was performed after Suzuki-Miyaura cross coupling reaction for 12 h. Scale bars: (b) 100 nm and (c) 250 nm.

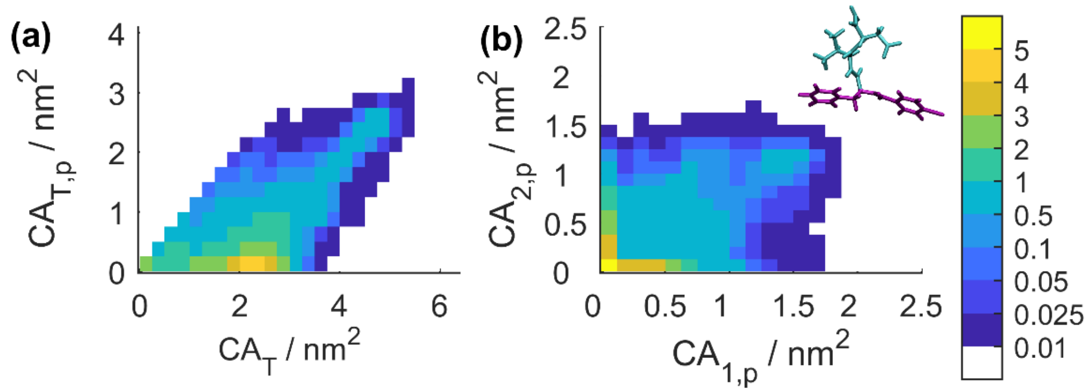

Figure S9. (a) Population maps of $CA_{T,p}$ against CA_T for tripeptide precursor $\mathbf{P_0}$. (b) Population maps of $CA_{2,p}$ against $CA_{1,p}$ for tripeptide precursor $\mathbf{P_0}$. The colorbar indicates the percentage of simulation time (note the non-linear increments). The inset shows the aromatic (purple) and peptide (cyan) regions from an arbitrarily selected snapshot of $\mathbf{P_0}$.

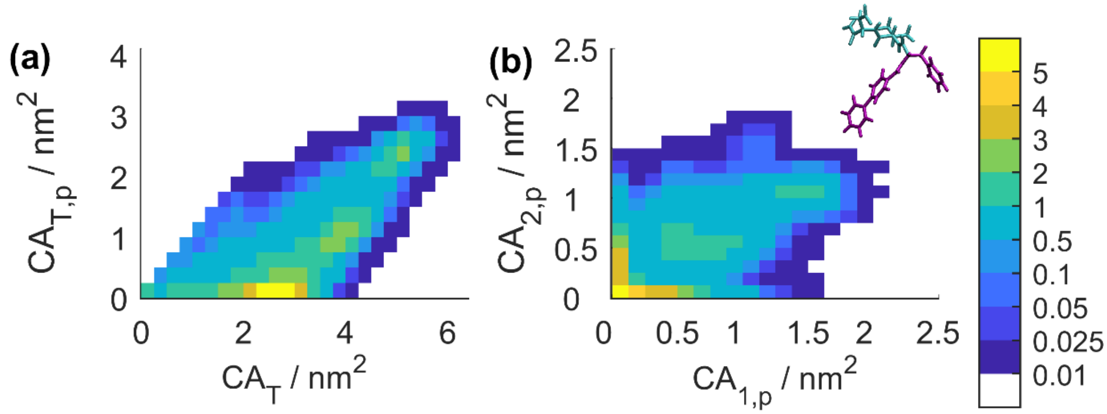

Figure S10. (a) Population maps of $CA_{T,p}$ against CA_T for tripeptide precursor $\mathbf{P_1}$. (b) Population maps of $CA_{2,p}$ against $CA_{1,p}$ for tripeptide precursor $\mathbf{P_1}$. The colorbar indicates the percentage of simulation time (note the non-linear increments). The inset shows the aromatic (purple) and peptide (cyan) regions from an arbitrarily selected snapshot of $\mathbf{P_1}$.

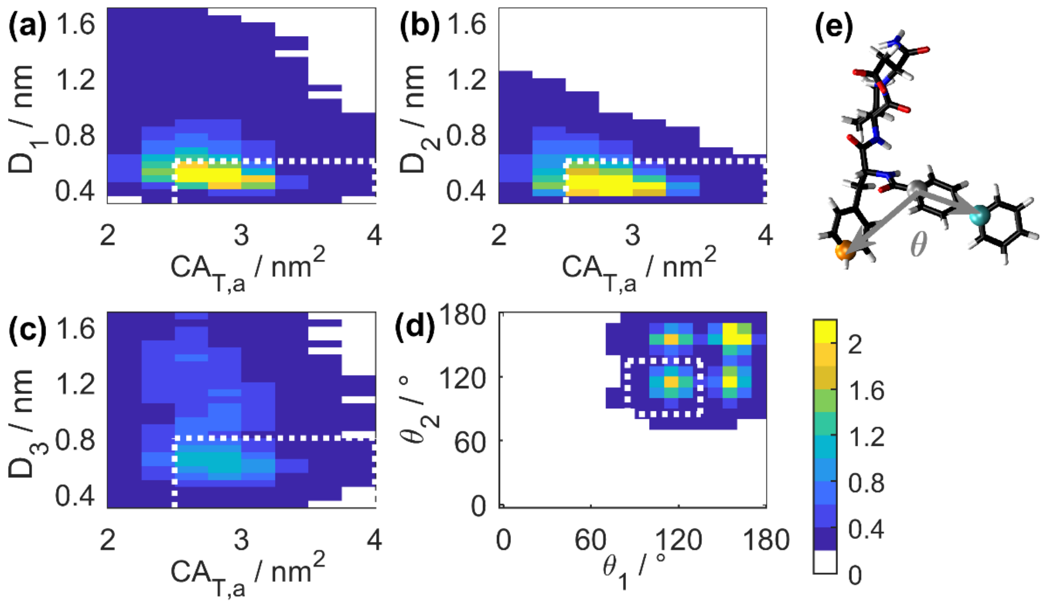

Figure S11. (a-c) Population maps of the distance between carbon atom pairs in tripeptide dimer against CA_T,a. The distances shown are those identified in Figure 3e; (d) the angle between the phenyl ring and the mutant ring defined as the angle between the three atoms shown in (e). For clarity all population maps for (a-d) are limited to conformations where both $CA_{1,a}$ and $CA_{2,a}$ are greater than 1 nm^2; (e) snapshot of $\mathbf{P_1}$ atoms used for angle calculation shown in (d). Dimer-conjugates with distances (a-c) and angles (d) contained within white dashed boxes were identified as those having the capacity to act as nucleation seeds for fibrillation.

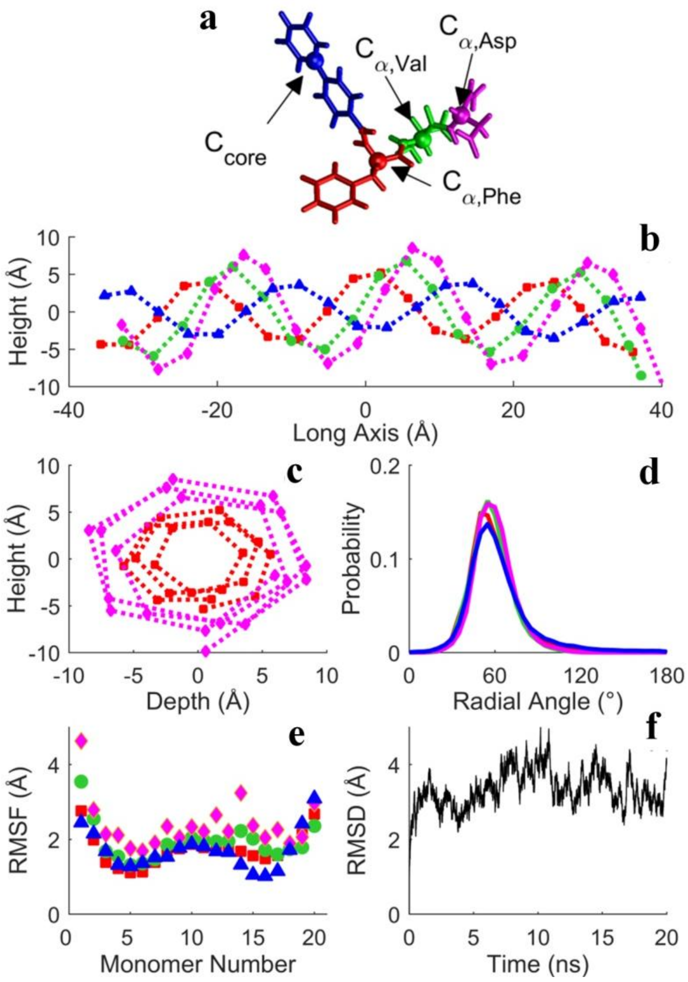

Figure S12. Monomer structure of tripeptide product $\mathbf{P_1}$ highlighting regions of the molecule forming the VDW surfaces in Figure 3b-f and the four atoms used for analysis presented in (b-f); (b) average position of the four atoms shown in (a) along the long axis of the fibre; (c) end view fibrillar aggregate showing of relationship between height and depth of C_α atoms of ASP and PHE; (d) distribution of radial angles of the four atoms shown in (a); (e) RMSFs of the four atoms shown in (a) along the length of the aggregate; and (f) RMSD including all non-hydrogen atoms of the fibrillar aggregate after removing for CoM motion. (b-f) are generated from data over the last 10 ns of the simulation.

Table 1. SASA of fibre and subregions compared to tripeptide $\mathbf{P_1}$ monomers in solution

	Total (Å^2)	Aromatic (Å^2)	PHE (Å^2)	VAL (Å^2)	ASP (Å^2)
**Monomer**	854	267	238	146	203
**Fibre**	377	72	100	49	155
**Exposed fraction**	0.441	0.269	0.422	0.335	0.766

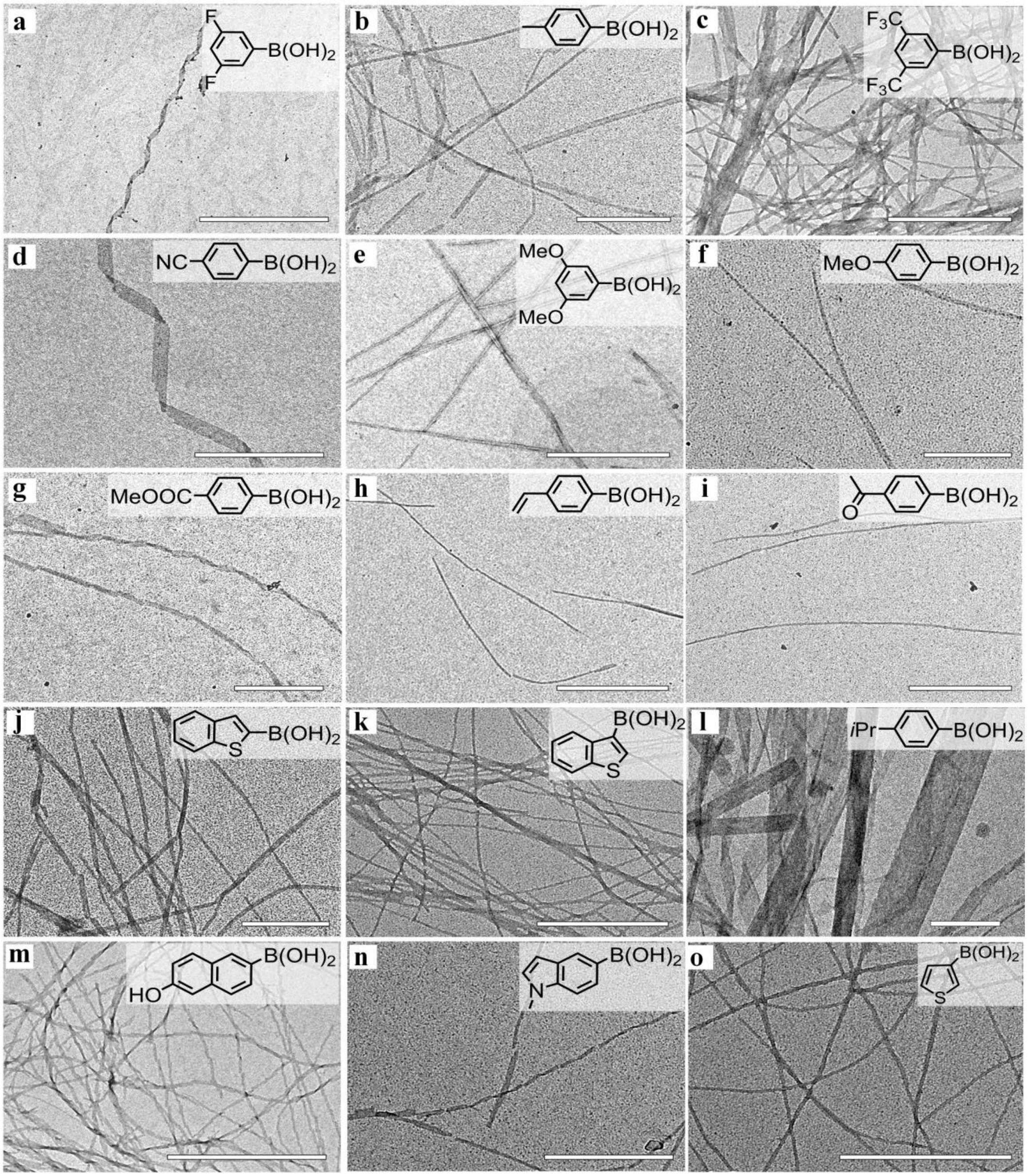

Figure S13. Negative-stained TEM images of supramolecular structures from self-assembly of product tripeptides. Suzuki–Miyaura cross coupling was conducted in the mixture of tripeptide precursor (**P_0**, 1.0 mM), aryl boronate (1.5 mM), and Na_2PdCl_2 (0.05 mM) in phosphate buffer (10 mM, pH 7.5). Scale bars: 500 nm.

Table S2. The log*P* of aryl groups of organic boronates listed in Figure 4. For example, the corresponding aryl groups of **1** and **2** are benzene and toluene, respectively. The values of log*P* were calculated from the on-line service provided by Molinspiration (https://www.molinspiration.com/cgi-bin/properties).

**#**	**1**	**2**	**3**	**4**	**5**	**6**	**7**	**8**	**9**
**log*P***	1.94	2.39	2.85	3.24	3.8	3.45	3.24	3.64	2.81
**#**	**10**	**11**	**12**	**13**	**14**	**15**	**16**	**17**	**18**
**log*P***	2.79	1.69	2.1	2.24	2.83	3.7	2.03	1.99	1.84
**#**	**19**	**20**	**21**	**22**	**23**	**24**	**25**	**26**	**27**
**log*P***	2.04	1.89	2.11	-1.56	-1.56	1.27	1.27	1.46	-2.13
**#**	**28**	**29**	**30**	**31**	**32**	**33**	**34**	**35**	**36**
**log*P***	0.63	0.81	1.28	1.73	1.13	0.9	0.96	0.96	1.6
**#**	**37**	**38**	**39**	**40**	**41**	**42**			
**log*P***	1.6	2.64	2.9	2.9	2.26	2.23			

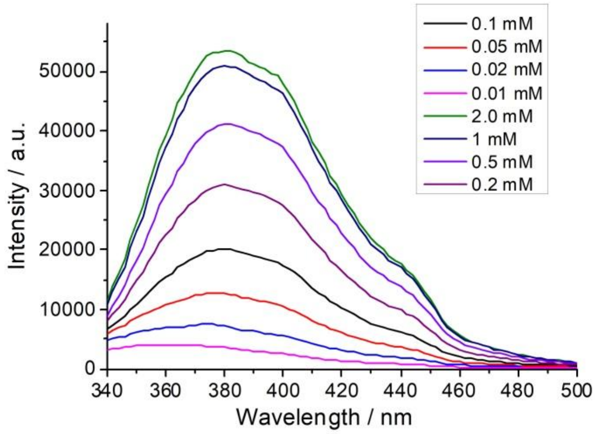

Figure S14. Concentration-dependent fluorescence emission spectra of a tripeptide product ($\mathbf{P_{37}}$): 0.01 mM, 0.02 mM, 0.05 mM, 0.1 mM, 0.2 mM, 0.5 mM, 1.0 mM and 2.0 mM. The excitation wavelength was 310 nm.

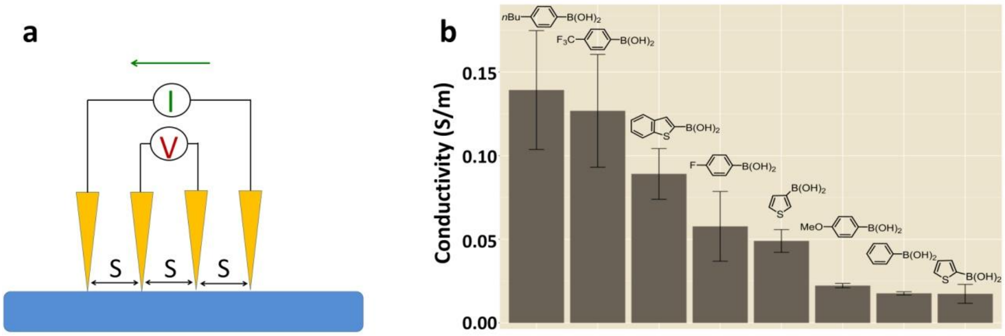

Figure S15. (a) Scheme of conductivity measurement using a four-point probe system. (b) Varying conductivity of self-assembled peptide films formed from Suzuki–Miyaura cross coupling between tripeptide precursor (**P_0**, 2.0 mM) and aryl boronates (2.5 mM), and Na_2PdCl_2 (0.1 mM). Self-assembled nanofiber films from hydrophobic and aromatic peptides, generally exhibit higher conductivity. The relatively low signal levels result in high measurement noise, hence the observed variance in measured values. Error bars indicate standard deviation of repeated measurements of conductivity values, bar height represents mean of measurement.

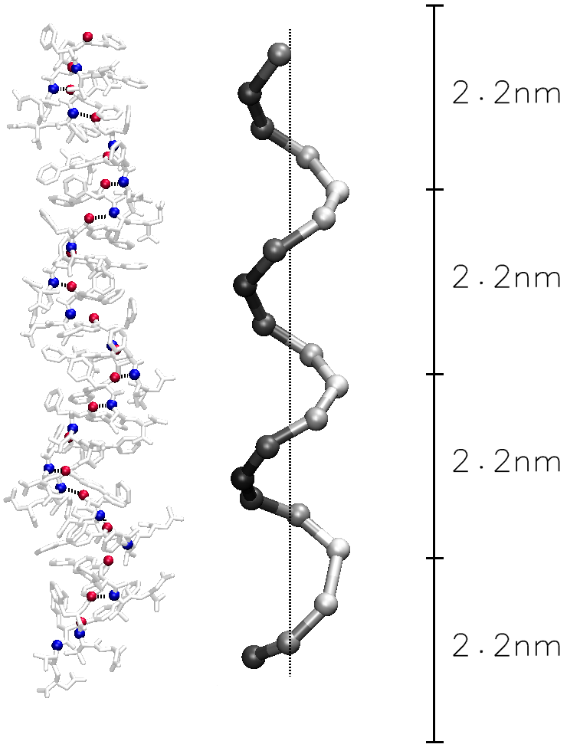

Figure S16. (left) Snapshot of the protofibre highlighting the hydrogen bonds (black dashed lines) between the nitrogen of the valine backbone (blue) and the terminal oxygen of phenylalanine (red) with the rest of the fibre shown in silver and (center) the helix generated by connecting the C_α of the valine residue with black and white indicating a deviation of 0.75 nm to the left or the right of the center of the helix (dashed line) and the horizontal and vertical bars showing the estimated width and pitch of the helix, respectively.
